# A Longitudinal fMRI Research on Neural Plasticity and Sensory Outcome of Carpal Tunnel Syndrome

**DOI:** 10.1155/2017/5101925

**Published:** 2017-11-16

**Authors:** Hao Ma, Yechen Lu, Xuyun Hua, Yundong Shen, Mouxiong Zheng, Wendong Xu

**Affiliations:** ^1^Department of Hand Surgery, Huashan Hospital, Fudan University, 12 Wulumuqi Middle Road, Shanghai 200040, China; ^2^Department of Central Laboratory, Jing'an District Centre Hospital, Shanghai, China; ^3^Department of Hand and Upper Extremity Surgery, Jing'an District Centre Hospital, Shanghai, China; ^4^Key Laboratory of Hand Reconstruction, Ministry of Health, Shanghai, China

## Abstract

Peripheral nerve compression is reported to induce cortical plasticity, which was well pictured by former researches. However, the longitudinal changes brought by surgical treatment are not clear. In this research, 18 subjects who suffered from bilateral carpal tunnel syndrome were evaluated using task-dependent fMRI and electromyography assessment before and after surgery. The third digit was tactually simulated by von Frey filaments. The results demonstrated that the pattern of activation was similar but a decreased extent of activation in the postcentral gyrus, inferior frontal lobe, superior frontal lobe, and parahippocampal gyrus after surgery was found. The correlation analysis showed a significant correlation between the decreased number of activated voxels and the improvement of EMG performance. This result implied a potential connection between fMRI measurement and clinical improvement.

## 1. Introduction

Carpal tunnel syndrome is mostly characterized by sensory deficit of fingers. Differing from the traditional views, peripheral neuropathy, even the mild entrapment such as CTS, could induce cortical plasticity [1, 2]. Most researchers focused on the plasticity of peripheral nerve compression. However, the longitudinal changes brought by surgical decompression have not been clearly pointed out. As the sole therapy after unsuccessful conventional treatment, surgery always plays an important role in the whole medical strategy of sensory recovery. We believed that the exploration on surgical impact of neural plasticity led to a better understanding of CTS. That might also be helpful in studying cortical plasticity induced by peripheral nerve decompression.

Napadow et al. assessed the functional and structural neuroplasticity in the somatosensory cortex when the fingers were electrically or mechanically stimulated [1, 3]. The cortical area of finger projection showed a trend of expansion, which was associated with median nerve compression at carpal level. This effect was reported to be reversed after acupuncture treatment for weeks, though the index finger was insensitive to the intervention [4, 5]. Considering the complexity and uncertainty of the neural mechanism of acupuncture, it was difficult to reason why the two fingers in one hand responded so differently in cortical plasticity. In another transcutaneous electrical nerve stimulation research, the pain-related cortical activations were reported to be significantly reduced [6]. It could be inferred that the pain-related cortical region was also involved in the sensory restoration process. Regretfully, it was still hard to speculate the changes of nerve decompression out of those indirect studies. The exact cortical reaction from nerve compression to nerve decompression would be an interesting topic.

In this study, we tracked 18 subjects who suffered bilateral CTS for an average of 6 months. We aimed to explore the longitudinal changes of cortical plasticity utilizing fMRI. In addition, the potential connection between plasticity and sensory recovery was also followed with interest.

## 2. Methods and Materials

### 2.1. Participants

A total of 18 subjects (3 male and 15 female) who suffered from bilateral CTS, aged 40–50 years old with a mean age of 45.2 ± 3.3 years, were enrolled in this study. The age and severity of disease assessed by the Boston Questionnaire were controlled for better homogeneity (detailed information is provided in Table 1). We only recruited patients with (1) determined diagnosis of CTS by physical and electrophysiological examination. The Phalen and Tinel tests are positive of the patients, and they demonstrated a “shaking out” movement to alleviate the numbness in the night. The nerve conduction studies (NCVSs) showed prolonged latency and reduced velocities of the median nerve. According to the electrophysiological classification by the American Association of Electrodiagnostic Medicine (AAEM), the inclusion criteria were (1) patients suffering from moderate or severe CTS; (2) patients who had neurological symptoms of more than 3 months and failed to respond to conservative treatment for more than 3 months; and (3) patients capable of receiving magnetic resonance scan without contraindication. Meanwhile, we excluded participants with (1) other concurrent peripheral neuropathies; (2) any of the contradictions of MR scan or metallic implants on the skull causing serious artifacts; (3) confirmed history of diabetes mellitus, cervical spondylopathy, rheumatic disease, multiple sclerosis, chronic pain, or cerebral diseases. A complete research protocol and written consent form were provided for all the participants. This research was approved by the medical ethical committee of the Medical Ethics Committee of the Huasha Hospital, Fudan University (Shanghai, China). All the process of the ethical work met the demands of the Helsinki declaration.

### 2.2. Clinical Assessment

We assessed the sensory function with EMG examination (Dantec Keypoint 4 workstation, http://www.natus.com/). The data was recorded from a sensory conduction examination. A routine EMG test usually consisted of motor and sensory tests. For sensory conduction examination, one circle-like surface electrode, used as recording electrode, was placed at the level of proximal interphalangeal joint. Another stimulus electrode (usually surface electrode) was fixed on the wrist level between the palmaris longus and flexor carpi radialis. The third surface electrode, which was also circle like and used as reference electrode, was placed around the distal interphalangeal joint of the finger. The motor measurements were not involved in this research. One irrelevant technician completed all the EMG tests and then transported the sensory conduction data to the analyzer.

### 2.3. Data Acquisition

The fMRI scan was carried out with a 3.0T scanner (MR750, GE Healthcare, USA). The participants underwent a protocol including one task-dependent block-design BOLD sequence and a FSPGR 3D-T1 sequence. The first fMRI scan was performed before the surgery, and the second one was performed 6 months after surgery, which allowed enough time for cortical plasticity and avoided the interference of surgical incision-related pain. Considering subjects' individual arrangement, a 2-week variation was allowed.

Foam padding was utilized to reduce head motion. For structural 3D-T1 imaging, we used the FSPGR sequence as follows: matrix size = 256 × 256, FOV = 256 × 256 mm, TR/TE = 8100/3.1 ms, FA = 8°, slice thickness = 1 mm, gap = 0 (isotropic voxel size = 1 × 1 × 1 mm), and TI (prepare time) = 450 milliseconds. For BOLD sequences, the parameters were as follows: sequence = GRE-EPI, interleaved scanning order, slice number = 43, matrix size = 64 × 64, FOV = 220∗220 mm, TR = 3000 ms, FA = 90°, slice thickness = 3.2 mm, gap = 0 (voxel size 3.4 × 3.4 × 3.2 mm^3^), and number of acquisitions = 120.

### 2.4. Experimental Paradigm

Each participant underwent the same protocol. They were required to complete one sensory task, in which their digit 3 of both hands was stimulated using the 300 g von Frey filament (length: 35.5 mm, diameter: 1.0 mm, and theoretical pressure: 292 g/mm^2^). Each session of the block design only involved a stimulus task of one hand. We utilized the models based on boxcar as our stimulus model during the task-dependent fMRI scan. We designed 6 cycles of ON-OFF. The whole scan starts with 12 seconds of dummy scan, which would automatically be discarded by the GE system. Then, an epoch of ON begins and lasts for 30 seconds. During this “ON” period, a 300 g von Frey filament was used to stimulate the finger pulp of the third digit of the subjects in a pseudorandom pattern, which in order to avoid sensory adaptation was brought by regular stimulus. Then, an epoch of “OFF” begins and lasts for 30 seconds, during which the stimulation was removed. One cycle lasts for 60 seconds and sequentially involves one ON and one OFF epoch. One complete session contains 6 cycles in all. The result of the first 3 cycles would be compared with the last 3 cycles in order to ensure uniformity. One single session of sensory stimulus task lasted for 6 min 12 sec in all. The whole scan of one subject mainly consisted of one 5 min-long FSPGR-T1 image and two 6 min 12 sec EPI block design images.

The stimulus area was located in the finger pulp, which was the most sensitive area of finger. The stimulus area was divided into 4 subregions. The stimulus task performed in each subregion lasted for a different period, and the frequency of tactile stimulus was also different. But the pattern of the lasting period and the frequency of stimulus together determined a specific protocol of sensory task, which was constant within subjects (presurgery and postsurgery) and across subjects. The homogeneity of the sensory task was controlled through utilizing a same technician and following a same protocol.

During the OFF period, the participants were instructed to keep calm and avoid unnecessary movements in the MRI scanner. During the sensory stimulation, the participants were required to relax their bodies with no muscle contraction. The whole scan lasted for about 12 minutes.

### 2.5. BOLD Data Preprocessing

A toolbox including Dpabi [7] (http://rfmri.org/DPABI) and SPM8 (http://www.fil.ion.ucl.ac.uk/spm/software/spm8/) was used for bold data processing. The first four dummy scans were already removed for signal equilibrium by the GE workstation. A slice timing procedure was applied for correcting the different acquisition times of each slice. Then, we realigned the functional volumes and corrected head motion by means of a six-parameter rigid body transformation [8]. Participants whose excessive head motions were above 3 mm in translational motion or 3° in rotational motion would be abandoned. One CTS patient's images were abandoned because of serious artifacts. The realignment procedure also generated a mean volume of the functional images. The T1 image was first coregistered to the previous generated mean volume, which adjusted the position matrix of the T1 image. Then, the T1 image was segmented into grey matter, white matter and cerebrospinal fluid, and a transformation matrix from individual space to the Montreal Neurological Institute space (MNI space). The functional volumes were then spatially registered to the MNI using deformation information obtained from the T1 image segmentation. The normalized functional volumes were smoothed with a Gaussian kernel of [6 6 6].

### 2.6. Statistical Analysis

The statistical analysis of fMRI data uses a mass-univariate approach based on general linear models (GLMs). According to the experimental paradigm, we specified the GLM design matrix and estimated the GLM parameters using classical or Bayesian approaches. Then, we interrogated the results using contrast vectors to produce a statistical parametric maps (T maps in this study). The threshold was set at *p* < 0.05 with FWE (family-wise error) correction. The significant activation area within groups through one sample *t*-test was reported and then binarized as a mask. A paired *t*-test was adopted to identify the difference of sensory stimulus response before and after surgery. The statistical comparison was carried out in the previous generated mask. The main analysis was performed within the boundary of the bilateral sensorimotor area. And the ROIs were extracted by WFU pickatlas tool (http://www.nitrc.org/projects/wfu_pickatlas/) and kept the same resolution and voxel size as the functional images.

### 2.7. Correlation Analysis

In addition to the fMRI analysis, we applied an extra correlation analysis of two measurements. The difference of both sensory latency and significant voxel number in the primary sensory area before and after surgery was calculated. And the ROIs were extracted by WFU pickatlas tool and kept the same resolution and voxel size as the functional images. A linear regression was done between the difference of voxel number in one hemisphere and the difference of sensory latency of the corresponding hand. The *R* square would be reported to indicate the goodness of fit.

## 3. Results

The average median nerve sensory velocity of the right hand was 38.75 ± 7.71 m/s before surgery and improved to 52.35 ± 8.23 m/s after surgery, while the left was 35.03 ± 7.13 m/s and 50.43 ± 8.97 m/s, respectively, before and after surgery. The average median nerve sensory latency of the right hand was 7.87 ± 1.87 m/s before surgery and decreased to 4.55 ± 1.94 m/s after surgery, while the left was 8.35 ± 1.65 m/s and 4.93 ± 2.05 m/s, respectively, before and after surgery. The sensory threshold of the right hand was 0.37 ± 0.23 g and improved to 0.04 ± 0.02 g after surgery, while the left was 0.37 ± 0.24 g and 0.06 ± 0.08 g, respectively, before and after surgery (Table 1). Paired *T*-test showed significant improvement of sensory conduction test after surgery.

**Figure 1:**
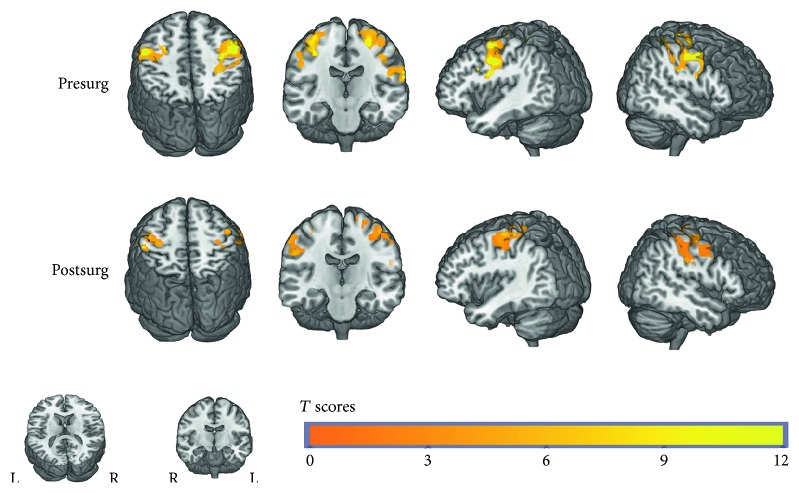
Activation pattern before and after surgery. The first row of the figure shows the activation pattern of the presurgery group through one-sample *t*-test. And the second row shows the pattern of the postsurgery group. The extent of the activation area of the presurgery group is much larger than that of the postsurgery group. Other activation areas have been hidden in the figure, which aims to minimize visual disturbance.

**Figure 2:**
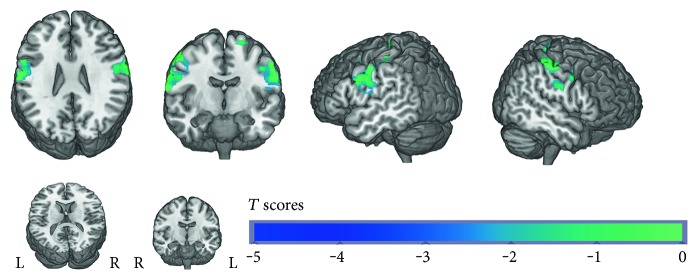
Difference of the activation pattern between presurgery and postsurgery. Areas including the bilateral postcentral gyrus, right parahippocampal, left inferior frontal lobe, and right superior frontal lobe show significantly decreased activation after surgery.

**Figure 3:**
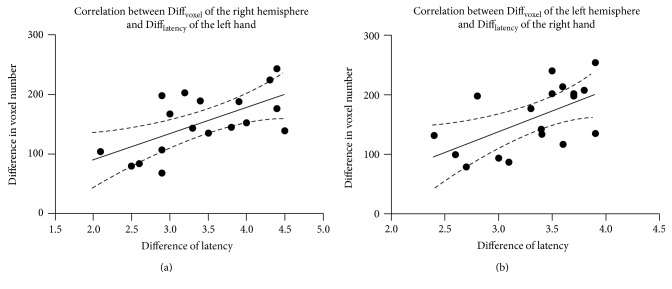
Results of the correlation analysis between fMRI measurement and clinical assessment. (a) The post-presurgery difference of activated voxels in the right postcentral gyrus is positively correlated with the post-presurgery improvement of sensory latency of the left-wrist median nerve. The *R* square is 0.39. (b) The post-presurgery difference of activated voxels in the left postcentral gyrus is also positively correlated with the post-presurgery improvement of sensory latency of the right-wrist median nerve. The *R* square is 0.34.

The one-sample *t*-test within group demonstrated that both presurgery and postsurgery groups showed significant activation in the postcentral gyrus. The pattern of activation was similar, but the extent of activation in the postsurgery group was smaller than that in the presurgery group (Figure 1). The paired *t*-test between groups revealed that the postsurgery group displayed a decrease of activation in the bilateral postcentral gyrus when sensory stimulus was applied. Despite of the primary sensory area, the difference of the activation area also involved the right parahippocampal gyrus, left inferior frontal lobe, and right superior frontal lobe (Figure 2, Table 2). We also analyzed the decreased number of activated voxels in the primary sensory area. And the improvement of sensory latency (postsurgery versus presurgery) was also calculated. The correlation analysis showed a significant correlation between the decreased number of activated voxels and the improvement of EMG performance (Figure 3), which implied a connection between fMRI measurement and clinical improvement. We compared the first half of the time series of block design with the last half. We found that the signal was steady across the time series and no significant difference was detected between the first 3 cycles and the last 3 cycles.

## 4. Discussion

Carpal tunnel syndrome is one common peripheral nerve entrapment, which is characterized as finger paresthesia. People used to hold the view that the recovery of peripheral neuropathy could only be influenced by peripheral factors, until Taylor et al. discovered the effect of peripheral nerve injury on cortical reorganization [9]. Some peripheral neural neuropathy, such as deafferentation or entrapment, results in specific changes of the functionally corresponding cortical area [10–12]. Our data provides a longitudinal research of the effects brought by surgical intervention in CTS. The study shows a significant reduction of the sensory projection area of the finger after surgery, which is mostly localized in the bilateral postcentral gyrus. The activation was also observed in the right parahippocampal gyrus, left inferior frontal lobe, and right superior frontal lobe after surgery. The correlation of the activated pattern with the clinical improvement is significant.

A previous study has already indicated that carpal tunnel syndrome was characterized by functional and structural neuroplasticity in primary somatosensory [1, 3, 13]. Compared to the cortical manifestations by the ulnar nerve innervated digit, the median nerve was amplified. In the fMRI study, while comparing to normal control subjects, the region of interest (ROI) cortical area representation digit 2 (D2) and digit 3 (D3) increased and the distance of the two areas decreased and overlapped in extent [1]. After acupuncture, the area was contracted and the distance of two ROI areas was increased and separated. The discrimination capacity of the digits increased [3, 5]. Another analysis revealed that the cortical grey matter volume in contralateral S1 reduced significantly, which might be associated with nerve conduction velocity and latency [14]. The researches above implied a probable connection between the neuroplasticity and functional deficits. The reduction of D2/D3 cortical separation distance was dramatically associated with paresthesia, reduction motor performance, and worse sensory discrimination accuracy. However, the knowledge about the longitudinal changes brought by surgical intervention was very limited.

In our study, both the presurgery and postsurgery groups showed significant activation in the postcentral gyrus. The statistical analysis demonstrated a reduced extend of activation in the primary sensory area after surgery. According to the further correlation analysis, this reduction of activation extent was positively correlated with the symptoms improved, which was denoted by the difference of latency before and after surgery.

The bilateral CTS patients were recruited in this research. All of the patients were right handed. Usually, the left hemisphere of the majority of the population is dominant for manual sensorimotor control [15–18]. We performed the correlation analysis of the left and right hands separately. Considering the influence of handedness, the results showed surprisingly little difference between the two hands. However, there is little direct evidence about the exact effect of handedness on sensory plasticity. The reason may be attributed to the smaller weights of sensory function in the handedness problem. The dominant side of sensory is not likely to be formed as motor function.

The postcentral gyrus is the main sensory representation area for being tactile [19–22]. Lesions in the postcentral gyrus induced characteristic somatosensory deficits, which symptoms including agraphesthesia, hypesthesia, and reduction of proprioception and fine touch [23, 24]. The changes of activation extent in the sensory cortex indicated a potential decrease of sensory afferentation. That might be caused by the reduction of numbness or paresthesia after surgical intervention. We tended to regard the increase of extend in SI as malplasticity during the median nerve compression. And the surgical decompression of the nerve reverses the process and makes the sensory cortex return to its normal boundary. The correlation study revealed a connection between fMRI measurement and clinical assessment. The improvement of the peripheral nerve has a potential influence on cortical sensory plasticity.

Another finding of the sensory stimulus study was the parahippocampal cortex (PHC) and dorsolateral prefrontal cortex. The PHC contributes to the network of brain regions participating in higher level cognitive processes, which involves visuospatial processing and episodic memory [25–28]. The dorsolateral prefrontal cortex is also comprehensively associated with working memory [29–32]. Because the tactile stimulus was performed in a pseudorandom pattern, we believed that the sensory stimulus, which was originally designed to eliminate the negative effect of sensory weariness, has trigged the memory function of subjects in the dull settings of the scanner.

Another problem worth discussing is the selection of measurement in the correlation analysis. Napadow et al. discovered that the digit 3 projection area in the primary somatosensory cortex demonstrated more extensive activation when sensory stimuli were applied. They reasoned that the increased extent of activation in the primary somatosensory may be due to CTS patients' persistent paresthesia and pain, which represented increased afferent input for the affected digits [1]. Although the CTS patients may feel more intense pressure after surgery, the missing of persistent paresthesia or pain is the main factor which affects the extent of activation. As is previously described, the von Frey filament has 20 levels of threshold. Sensory threshold is a kind of discontinuous variable and it is not suitable for regression analysis. So, we chose sensory latency of the EMG test instead.

The current study demonstrated a longitudinal change of sensory plasticity brought by surgical intervention. The changes of neuroplasticity were correlated with the improvement of symptoms. That revealed a potential connection between clinical outcome and cerebral functional reorganization.

## 5. Conclusion

CTS patients showed a decreased extent of activation in the postcentral gyrus, inferior frontal lobe, superior frontal lobe, and parahippocampal gyrus after surgical intervention. The changes of activated voxels in the primary sensory area showed a significant correlation with the improvement of EMG performance. This study provided a longitudinal view of cortical plasticity brought by surgical decompression. And the result also implied a potential connection between fMRI measurement and clinical improvement.

## 6. Limitation

Although correlation analysis between voxel size and brain activity revealed significant results, this result was based on the data smoothed by a relatively large kernel. Magnitude of activity/deactivity affects the size of the area. Tests with much smaller kernel should be brought into consideration in smooth procedure.

## Figures and Tables

**(a):** 

Carpal tunnel syndrome (*n* = 18, 15 female)	
Ages (years)	45.2 ± 3.3
Interval before scan (months)^∗∗^	5.0 ± 2.8
Boston Questionnaire (symptom)	2.29 ± 0.46
Boston Questionnaire (daily life)	2.01 ± 0.63

**(b):** 

Nerve conduction study	Presurgery	Postsurgery	*p* value
Median nerve sensory velocity (m/s)	Left: 35.03 ± 7.13	Left: 50.43 ± 8.97	*p* < 0.05
	Right: 38.75 ± 7.71	Right: 52.35 ± 8.23	*p* < 0.05
Median nerve sensory latency (m/s)	Left: 8.35 ± 1.65	Left: 4.93 ± 2.05	*p* < 0.05
	Right: 7.8 ± 1.87	Right: 4.55 ± 1.94	*p* < 0.05
Sensory threshold (g)	Left: 0.37 ± 0.24	Left: 0.06 ± 0.08	*p* < 0.05
	Right: 0.37 ± 0.23	Right: 0.04 ± 0.02	*p* < 0.05

Data is shown as mean ± SD; ^∗∗^interval before scan means the time period between determined diagnosis and fMRI scan.

**Table 2:** Paired *t-*test results of activation pattern of sensory stimulus task between presurgery and postsurgery.

Region	MNI coordinate			Voxels in cluster	*p* value (FDR corrected)
Right parahippocampal	30	0	−30	110	*p* < 0.05
Left inferior frontal	−48	36	18	245	*p* < 0.05
Right superior frontal	30	0	66	75	*p* < 0.05
Right postcentral	52	−14	35	86	*p* < 0.05
Left postcentral	−48	−11	33	74	*p* < 0.05
